# A comparison of the outcomes of dexmedetomidine and remifentanil with sufentanil-based general anesthesia in pediatric patients for the transthoracic device closure of ventricular septal defects

**DOI:** 10.1186/s13019-021-01498-8

**Published:** 2021-04-23

**Authors:** Ling-Shan Yu, Wen-Peng Xie, Jian-Feng Liu, Jing Wang, Hua Cao, Zeng-Chun Wang, Qiang Chen

**Affiliations:** 1 Department of Cardiac Surgery, Fujian Maternity and Child Health Hospital, Affiliated Hospital of Fujian Medical University, Fuzhou, China; 2 Fujian Key Laboratory of Women and Children’s Critical Diseases Research, Fujian Maternity and Child Health Hospital, Fuzhou, China; 3 Fujian Branch of Shanghai Children’s Medical Center, Fuzhou, China; 4 Fujian Children’s Hospital, Fuzhou, China; 5 Department of Cardiovascular Surgery, Union Hospital, Fujian Medical University, Fuzhou, China

**Keywords:** Cardiac anesthesia, CHD, Septal defects, Cardiac intervention

## Abstract

**Objective:**

To compare the safety and efficacy of dexmedetomidine and remifentanil with sufentanil-based general anesthesia for the transthoracic device closure of ventricular septal defects (VSDs) in pediatric patients.

**Methods:**

A retrospective analysis was performed on 60 children undergoing the transthoracic device closure of VSDs from January 2019 to June 2020. The patients were divided into two groups based on different anesthesia strategies, including 30 cases in group R (dexmedetomidine- and remifentanil-based general anesthesia) and 30 cases in group S (sufentanil-based general anesthesia).

**Results:**

There was no significant difference in preoperative clinical information, hemodynamics before induction and after extubation, postoperative pain scores, or length of hospital stay between the two groups. However, the hemodynamic data of group R were significantly lower than those of group S at the time points of anesthesia induction, skin incision, thoracotomy, incision closure, and extubation. The amount of intravenous patient-controlled analgesia (PCA), the duration of mechanical ventilation, and the length of the intensive care unit (ICU) stay in group R were significantly less than those in group S.

**Conclusion:**

Dexmedetomidine combined with remifentanil-based general anesthesia for the transthoracic device closure of VSDs in pediatric patients is safe and effective.

## Introduction

Ventricular septal defects (VSDs) are a common congenital heart disease. Traditional surgical repair with cardiopulmonary bypass is associated with great trauma, a certain percentage of postoperative complications and a long hospital stay [1]. With the development of minimally invasive techniques in cardiac surgery in recent years, the transthoracic device closure of VSDs with small incisions under echocardiographic guidance has increased gradually, especially in China. This approach has the advantages of a small incision and a short operating path, guidance by echocardiography, quick recovery, and a short hospital stay [2]. The corresponding requirements for the cardiac anesthesia strategy come into play: choosing the appropriate anesthetic regimen for tracheal extubation as soon as possible, shortening the length of the intensive care unit (ICU) and hospital stay, and finally reducing the medical cost [3]. Most studies have shown that fast-track cardiac anesthesia for children is feasible and safe, and many anesthesiologists have their own clinical experience in guiding the implementation of the corresponding anesthesia strategy in pediatric cardiac surgery [4–6]. We also reported our experience with the use of remifentanil-based general anesthesia for the transthoracic device closure of VSDs through small incisions under echocardiographic guidance [7]. In this study, we tried to compare the safety and efficacy of another anesthesia strategy of dexmedetomidine and remifentanil with those of the strategy of sufentanil-based general anesthesia for the transthoracic device closure of VSDs in pediatric patients.

## Materials and methods

The present study was approved by the ethics committee of our university (grant no. 2009024) and adhered to the Declaration of Helsinki. Because the use of dexmedetomidine in children is off-label, we mentioned this off-label use in the consent form and communicated with the patient’s parents. Finally, written informed consent about this study was obtained from the parents.

### Clinical data

Through preliminary study results, the alpha value was set as 0.05, and the statistical power was set as 0.9. Then, considering a certain exclusion rate, it could be calculated that the minimum sample size needed to complete the study was 60. A total of 188 consecutive children with VSD who underwent transthoracic device closure from January 2019 to June 2020 were retrospectively analyzed. According to the different anesthesia strategies administered and the propensity matching score (1:1), a total of 60 children who met the inclusion criteria were matched, including 30 cases in group R (dexmedetomidine- and remifentanil-based general anesthesia) and 30 cases in group S (sufentanil-based general anesthesia). Since this study was not a prospective study but a retrospective study, we tried our best to select matching patients, who accounted for only a small part of the patients in the same period. Due to the differences in the patient’s condition and the surgeon’s experience and technology, the duration of the operation was correspondingly different. These factors might affect the choice of anesthesia protocol. Therefore, the choice of anesthesia protocol was made by the anesthesiologist and the surgeon, and the anesthesiologist took the main responsibility.

The inclusion criteria included transthoracic echocardiography (TTE) diagnosed as isolated VSD and evidence of significant left to right shunts, an adequate margin up to the indications of device closure of VSD, and no obvious anesthesia or VSD device closure contraindication. Exclusion criteria included other cardiac abnormalities requiring concurrent surgical correction, device closure failure, severe pulmonary hypertension, severe pneumonia and respiratory insufficiency, chronic cardiac, hepatic and renal insufficiency, and age below 1 year. All patients completed routine preoperative examinations, and the relevant data are shown in Table 1. Some patients had delayed growth and development and easily suffered from respiratory tract infections or pneumonia.

**Table 1:** General preoperative conditions of children

	Group R (***n*** = 30)	Group S (***n*** = 30)	***P*** value
**Age (years)**	2.2 ± 0.7	2.3 ± 0.8	0.57
**Gender (male/female)**	16/14	15/15	0.62
**Body weight (kg)**	13.9 ± 2.4	14.0 ± 2.4	0.77
**Size of VSD (mm)**	4.2 ± 0.9	4.3 ± 0.8	0.80
**LVEF (%)**	68.5 ± 2.5	67.8 ± 3.1	0.78
**Qp/Qs**	1.8 ± 0.3	1.9 ± 0.4	0.81

### Dexmedetomidine- and remifentanil-based general anesthesia

Patients routinely fasted for 4 h and were forbidden to drink for 2 h before surgery and were injected with 0.05 mg/kg midazolam intramuscularly before entering the operating room. ECG and peripheral blood oxygen saturation monitoring were performed, and 5% glucose solution was infused intravenously. Arterial blood pressure and central venous pressure were monitored by artery and subclavian vein puncture. Anesthesia induction included the intravenous injection of 0.05 mg/kg midazolam, 1.0 μg/kg remifentanil and 0.5 mg/kg cisatracurium besylate. Then, endotracheal intubation was performed, followed by connection of the tube to the anesthesia machine that provided mechanical ventilation. The ventilation mode was pressure control mode. Nasopharyngeal temperature was monitored, and the body temperature was maintained above 36.5 °C. Arterial blood gas was analyzed to assess ventilation conditions. The maintenance of anesthesia was intravenous pumping of 0.1–0.5 μg/kg/min remifentanil and 0.5 μg/kg/h dexmedetomidine and inhalation of 2–3% sevoflurane until the procedure was over. Dexmedetomidine was continually pumped until the endotracheal intubation was removed.

### Sufentanil-based general anesthesia

The preparation before anesthesia induction was the same as that in group R. Induction of anesthesia consisted of the intravenous injection of 0.05 mg/kg midazolam, 1 μg/kg sufentanil and 0.5 mg/kg cisatracurium besylate. Anesthesia maintenance was performed by sufentanil 0.1–1 μg/kg/h intravenous pumping and sevoflurane inhalation 2–3%.

### Surgical procedure

The patient was placed in a supine position, and a transesophageal echocardiography (TEE) probe was placed into the esophagus. An incision of approximately 2–3 cm at the lower sternum was made. Then, the pericardium was opened to expose the free wall of the right ventricle, and heparin was administered intravenously at 1 mg/kg. A surgery purse was made on the free wall of the right ventricle after the puncture site was confirmed under TEE guidance. Under continuous TEE guidance, a delivery pathway was performed to establish the right ventricular - VSD - left ventricular delivery track, which was sent to the occluder. The left and right dishes of the occluder were released to close the VSD. After confirming the proper position of the occluder with no significant residual shunt, no aortic valve regurgitation and no atrioventricular block, the occluder was released, and the delivery sheath was withdrawn [2]. Then, the patients were sent to the ICU with endotracheal intubation for further monitoring and treatment.

### Postoperative management

Postoperative management of all patients was completed by a unified ICU medical team, who decided the treatment plan and when to remove endotracheal intubation and discharge from the ICU according to the patient’s actual situation. The endotracheal intubation was removed when the patient woke up naturally and respiratory function was restored. The patient-controlled analgesia (PCA) method was used to perform intravenous analgesia for all patients. A standard protocol for 2.0 μg/kg sufentanil, 0.2 mg/kg tropisetron and 100 ml physiological saline was continuously pumped at a rate of 2 ml/h, and the dosage was adjusted or stopped according to the patient’s condition. If the pain score of the patient was greater than 4, the ICU doctor or nurse could press an intravenous PCA. Other postoperative care and medications were the same in both groups.

### Data collection

Data were collected and used to conduct the statistical analysis, which included: (1) general preoperative conditions of the children, perioperative hemodynamic index containing mean arterial blood pressure (MAP) and heart rate (HR) at time points before and after endotracheal intubation, skin incision, thoracotomy, incision closure, extubation and 1 h and 4 h after extubation; (2) a pain behavior scale (face, legs, activity, crying, and consolability (FLACC)) was used to score at 1 h and 4 h after extubation, and the scores ranged from 0 to 10, where a higher score corresponded to more severe pain [8]; (3) the amount of intravenous PCA for each group; (4) the duration of mechanical ventilation, the length of ICU stay, and the length of hospital stay. Objective data were collected from the hospital’s medical record system, while subjective data were collected by an independent investigator.

### Statistical analysis

All data were entered into Excel and analyzed by IBM SPSS 20.0 statistical software. The propensity matching score was used for case matching, and the case and control groups were matched 1:1. The continuous data were checked and confirmed to conform to a normal distribution by a test for normality and were statistically analyzed by a paired samples t-test. The chi-square test was used to compare categorical variables between the two groups. A *P* value less than 0.05 was defined as significant.

## Results

The device closure of VSDs was successful in all patients. As shown in Table 1, there was no significant difference in preoperative general conditions between the two groups (*P* > 0.05). Table 2 shows that the MAP and HR in group R were significantly lower than those in group S at the time points of endotracheal intubation, skin incision, thoracotomy, incision closure, and extubation (*P* < 0.05). The hemodynamic indexes and pain scores of the two groups at 1 h and 4 h after extubation showed no significant differences (*P* > 0.05, Tables 2 and 3), but the amount of postoperative opioids of group R was significantly less than that of group S (*P* < 0.05, Table 3). Table 4 indicates that the duration of mechanical ventilation and the length of ICU stay in group R were significantly less than those in group S (*P* < 0.05). There was no significant difference in the length of hospital stay between the two groups (*P* > 0.05). No severe complications associated with device closure or anesthesia-related complications occurred in either group. No vasoactive drugs were needed during the operation.

**Table 2:** Intraoperative and postoperative hemodynamic data

	Group R (***n*** = 30)	Group S (***n*** = 30)	***P*** value
**Preinduction MAP (mmHg)**	76.2 ± 3.3	76.3 ± 2.4	0.92
**Preinduction HR (beats/min)**	103.3 ± 6.1	104.0 ± 7.2	0.67
**After intubation MAP (mmHg)**	73.1 ± 2.1	76.9 ± 2.5	0.00
**After intubation HR (beats/min)**	92.7 ± 4.4	98.1 ± 7.0	0.00
**Skin incision MAP (mmHg)**	76.4 ± 2.4	78.1 ± 2.0	0.00
**Skin incision HR (beats/min)**	94.2 ± 4.6	100.7 ± 7.1	0.00
**Thoracotomy MAP (mmHg)**	78.4 ± 3.0	80.9 ± 2.5	0.00
**Thoracotomy HR (beats/min)**	95.2 ± 4.8	102.9 ± 7.0	0.00
**Incision closure MAP (mmHg)**	75.3 ± 2.0	76.6 ± 1.6	0.00
**Incision closure HR (beats/min)**	93.1 ± 3.6	99.5 ± 7.1	0.00
**Extubation MAP (mmHg)**	76.5 ± 3.5	79.6 ± 3.0	0.00
**Extubation HR (beats/min)**	94.3 ± 4.5	99.6 ± 6.9	0.00
**After extubation 1 h MAP (mmHg)**	76.8 ± 3.5	76.7 ± 3.4	0.86
**After extubation 1 h HR (beats/min)**	97.8 ± 4.9	99.2 ± 5.1	0.20
**After extubation 4 h MAP (mmHg)**	77.8 ± 3.3	78.7 ± 1.8	0.17
**After extubation 4 h HR (beats/min)**	100.9 ± 5.0	101.9 ± 5.4	0.27

**Table 3:** Pain score (FLACC score) and the amount of PCA at different time point after extubation

	Group R (***n*** = 30)	Group S (***n*** = 30)	***P*** value
**The FLACC score at 1 h after extubation**	0.2 ± 0.5	0.2 ± 0.4	0.77
**The FLACC score at 4 h after extubation**	1.1 ± 0.3	1.1 ± 0.2	0.57
**The amount of PCA at 1 h after extubation (ug)**	0.4 ± 0.1	0.7 ± 0.1	0.02
**The amount of PCA at 4 h after extubation (ug)**	2.0 ± 0.3	2.4 ± 0.4	0.04

**Table 4:** The different clinical results in both groups

	Group R (***n*** = 30)	Group S (***n*** = 30)	***P*** value
**The duration of mechanical ventilation (h)**	0.4 ± 0.1	1.4 ± 0.1	0.00
**The length of ICU stay (h)**	4.3 ± 0.2	9.7 ± 0.6	0.00
**Hospital stay (days)**	3.3 ± 0.5	3.5 ± 0.5	0.11

## Discussion

The transcatheter device closure of VSDs requires expensive equipment and treatment costs, and both doctors and patients need to be exposed to X-ray radiation. The operation process is relatively complicated, and the incidence of atrioventricular block is relatively high, which is difficult for many families to accept. To the best of our knowledge, the device closure of VSDs is not advisable in the opinion of some scholars, mainly because of the occurrence of complete atrioventricular block [9]. In fact, the transthoracic device closure of VSDs has been performed in many centers in China, and their clinical experience has been reported with a low incidence of associated atrioventricular block [2]. Increasing numbers of Chinese patients are opting for the transthoracic device closure of VSDs.

Long-lasting opioids are used in cardiac surgery, which may result in the postoperative depression of respiratory function and prolong the duration of mechanical ventilation and the length of ICU stay. Then, the postoperative complications are corresponding increased, and the length of hospital stay is also prolonged. At present, the commonly used clinical anesthesia strategy is to choose the appropriate anesthesia methods and drugs and to provide the appropriate depth of anesthesia that can not only meet the needs of surgery but also facilitate the early recovery of postoperative autonomous breathing and shorten the duration of mechanical ventilation and the length of ICU stay. Combined with the implementation of an appropriate anesthesia strategy for patients who underwent the transthoracic device closure of VSDs, this approach could be more conducive to reducing postoperative complications and shortening both the recovery process and the length of hospital stay.

In this study, sufentanil, which has a high plasma protein binding rate, the strongest analgesic effect, and slight cardiovascular effects, was used in group S. However, its clearance half-life is approximately 150 min, and it is associated with an increase in the intraoperative dose, an increase in the time of postoperative sobriety and delayed extubation [10]. A prospective study by Groesdonk et al. concluded that the use of short-acting opioids and inhaled anesthetics were safe in cardiac surgery [11]. In recent years, the application of remifentanil-based general anesthesia has gradually increased and proven to be safe and effective [12, 13]. Remifentanil is a short-acting opioid receptor agonist that works fast, and the effect wears off quickly after stopping the medicine. After stopping infusion for 3–5 min, spontaneous breathing resumes, which does not change with infusion time or age. Strong analgesic effects, stable hemodynamics, and good controllability make remifentanil especially suitable for fast recovery in children who undergo the transthoracic device closure of VSDs [14].

Lison and his colleagues found that patients who used remifentanil had a higher pain score 1 h after surgery than those who used sufentanil [15]. Because remifentanil can induce hyperalgesia when discontinued, in the past, it was believed that infants and children had a slow response to pain, and postoperative analgesia was often neglected. However, the pain stress response in adults can also be reflected in children [16]. In addition, in the recovery period of general anesthesia, incision pain and endotracheal intubation stimulation may cause cough, delirium, restlessness, and chills in children, leading to an accelerated heart rate, vascular resistance and increased myocardial oxygen consumption. Therefore, further research is needed to focus on postoperative pain treatment and to formulate a more precise analgesic program.

Dexmedetomidine is a type of α2 adrenal agonist with high efficiency and high selectivity. It has the effects of sedation, analgesia, anti-anxiety and anti-sympathetic nerve and little respiratory inhibition. It can obviously reduce the amount of anesthetic during the perioperative period, effectively relieve the stress response and ease postoperative pain. Studies on the application of dexmedetomidine in cardiovascular surgery have been increasing recently, and most of them show that dexmedetomidine has the effect of myocardial protection and hemodynamic stabilization [17–19]. Dexmedetomidine has been increasingly used in pediatric surgery in recent years, but it is still in the exploratory stage [20–22]. Related studies have indicated that the pharmacokinetic parameters of dexmedetomidine in children are basically similar to those in adults, but the clearance rate is relatively low because of the immature clearance path in children less than 1 year old [23]. Cheng and his team found that dexmedetomidine infusion in pediatric cardiac surgery with 0.25–0.75 μg/kg/h showed little variability in heart rate and blood pressure [24]. In our study, the infusion dose of dexmedetomidine in group R was 0.5 μg/kg/h, and combined with remifentanil and sevoflurane, the hemodynamics were stable. Dexmedetomidine and remifentanil did not cause bradycardia or hypotension without rapid infusion [25].

Both groups of children in our study were given intramuscular midazolam before entering the operating room to make them quiet and to produce amnesia. The duration between the patient’s admission to the operating room and undergoing heparinization was more than 1 hour, so local bleeding or hematoma could be avoided. The patients inhaled sevoflurane; blood gas solubility was low, and the depth of anesthesia was controllable, had no stimulation of the respiratory tract, and had a protective effect on acute myocardial injury [26]. Relevant studies confirmed that the use of cisatracurium as a muscle relaxant in pediatric cardiac surgery would not affect the postoperative duration of mechanical ventilation and the length of ICU stay [27]. Cisatracurium was selected as a muscle relaxant in anesthesia induction in both groups, and no additional dose was added in the operation due to the short procedural time.

Table 2 shows that the MAP and HR in group R were significantly lower than those in group S at the time points of endotracheal intubation, skin incision, thoracotomy, incision closure and extubation, demonstrating that dexmedetomidine combined with remifentanil could inhibit the stress response more effectively than sufentanil for the transthoracic device closure of VSDs in pediatric patients. While the hemodynamic indexes and pain scores of the two groups at 1 h and 4 h after extubation showed no significant difference, the amount of postoperative opioids in group R was significantly less than that in group S, which suggests that dexmedetomidine could provide effective and additional postoperative analgesia. Such results are consistent with the study results of Czaja et al., and it could be concluded that dexmedetomidine could provide certain sedative and analgesic effects and prevent and improve remifentanil hyperalgesia during the postoperative recovery stage [28]. Nguyen et al. concluded that dexmedetomidine’s unique properties made it an ideal sedative for patients undergoing cardiac surgery in a retrospective study because it had no respiratory suppression and had the potential to reduce opioid use and anti-sympathetic effects, which might shorten the duration of extubation and ICU observation [29]. Hashemian et al. showed that continuous intravenous pumping of dexmedetomidine at a rate of 0.5 μg/kg/h did not increase the recovery duration of ICU patients, which was also confirmed by our study [30].

There were some limitations in this study. This was not a prospective randomized controlled study but a retrospective study. The selection of cases might have a certain bias, which would also affect the statistical efficiency of this study. This was a limitation that could not be controlled by the retrospective study. In addition, subjectivity or empiricism in the treatment of children could not be ruled out and needs to be further evaluated by future prospective studies. However, we still believe that the results of this article have some clinical significance. Second, the sample size of this study was relatively small, and it was a single-center study, so the conclusion might be one-sided to some extent. Therefore, we look forward to completing multicenter, larger-sample studies in the future to confirm the reliability of our conclusions.

## Conclusion

In conclusion, dexmedetomidine- and remifentanil-based general anesthesia were used in the transthoracic device closure of VSDs in pediatric patients with early extubation and a shorter ICU stay. This anesthetic strategy is safe and effective and is worthy of clinical promotion.

## Data Availability

Data sharing not applicable to this article as no data sets were generated or analyzed during the current study.

## Abbreviations

VSD: Ventricular septal defect
ICU: Intensive care unit
MAP: Mean arterial blood pressure
HR: Heart rate
LVEF: Left ventricular ejection fraction
